# Supplementary material for: “If I’ve got latent TB, I would like to get rid of it”: Derivation of the CARD (Constraints, Actions, Risks, and Desires) Framework informed by South African healthcare worker perspectives on latent tuberculosis treatment
Source: PLoS One. 2021 Aug 18;16(8):e0254211. doi: 10.1371/journal.pone.0254211 (PMC8372902; doi:10.1371/journal.pone.0254211)
Supplement: S1 File — (DOCX) [file pone.0254211.s001.docx]

**SAFE Qualitative Study Interview Guide**

**Introduction**

*You have been randomly selected for an in-depth interview that will take between 30-60 minutes. The purpose of this interview is to document your experiences about TB transmission risk and explore the barriers to implementing successful screening programmes. During this interview, we wish to learn about your knowledge, attitudes and practices regarding tuberculosis transmission and screening. We hope to understand your needs and the best way to bring information to you, as well as barriers to seeking medical care. The information you provide will be used to improve TB screening.*

**Interview guide organized by topic areas:**

1. **PERSONAL RISK**

Tell us about your role in hospital? (would confirm department they work in)

What do you do from day to day? How long have you worked here?

What kinds of patients do you see?

Where do you see patients with TB? Are they often coughing? Where do you see patients with TB? Are they often coughing?

What do you do for patients who may have TB?

When you discharge them/ when you give them medication, is there any counselling involved?

Do you enjoy working here?

Are there times where you feel concerned about your own risk of contracting TB?

When, Why is that, What happened? What about your life makes you concerned?

Do you think you’ve been exposed to TB anywhere else other than the hospital?

Has it ever happened that a HW acquired TB? Tell me what happened?

1. **SYSTEM CHALLENGES**

What measures do you take to prevent yourself from contracting TB?

Do you do this for all patients or only for patients being evaluated for TB? How do you decide?

Do you ever try to avoid certain areas in the hospital or caring for certain patients because you’re afraid of contracting TB?

Are you aware of any measures that are in place to prevent TB transmission at Tygerberg Hospital?

Can you give examples?

Are you aware of any other measures that could prevent TB transmission in hospitals? What more could be done at Tygerberg?

What would it take to implement recommended TB infection control measures in the hospital?

Where does the information you have about TB come from?

Where did you learn about TB, infection control, your risk?

Tell me more about this?

How would you improve or change this?

Has training ever been repeated?

1. **ACTIVE VS LATENT TB**

Have you heard of the concept or term ‘latent TB’ or ‘TB infection’?

What is your understanding of this?

When, what have you heard others say about this? In what context, who?

How do you understand the difference between latent and active TB?

Does it mean you have TB?

Does it mean you are protected against getting active TB?

Who gets Latent TB and who gets Active TB?

Do you have any concerns about latent TB? / Is latent TB something that worries you?

Explore the term reactivation: Are you sick when you have latent TB? Can you become ill if you have latent TB?

Explore the term reinfection: Can you get TB again once you’ve had it?

1. **SCREENING**

Have you ever been tested for TB either in hospital or elsewhere?

*If they move straight to mentioning SAFE study test then clarify if they have ever been tested for active TB disease?*

What was done- X-ray, sputums? Was this due to symptoms you had or for screening?

Have you ever been tested for latent TB?

You had a blood test done as part of this SAFE study. What is your understanding of that?

Do you know if you had a positive or negative QFT?

Has it changed the way you think about TB and your risk/ your practices? How?

If QFT POS: Did occupational health follow up with you after you had your results? What happened?

Is screening for active TB something that the hospital should offer? Why/why not?

Do you think HCW would want to be screened?

What are the barriers to implementing this type of screening?

Is it possible to treat latent TB? Have you heard that treatment for latent TB may be effective or beneficial?

What makes you think that?

Would you take preventive therapy for latent TB infection if it were offered to you?

Why/Why not?

Are you worried about side effects? Which ones?

Are you worried about the costs?

Are you worried it would not be effective?

Have you heard that it is useless to test or treat South African HWs for latent TB since everyone is positive?

Have you ever heard that treating latent TB infection might remove the protective effects of the infection itself?

If you were told that taking treatment for latent TB would reduce your risk of developing active TB by 80%, would this make you more likely to take preventive therapy?

If there were a shorter regimen that you have to take for 1-3 months, would you be more willing to take the treatment?

Do you think the hospital should offer latent TB screening?

Have you worn a CO2 monitor as part of the SAFE study? How did that go?

Could CO2 monitoring be used as a potential strategy to identify times where there was a higher risk for acquiring TB?

Any questions? Anything else you would like to add?
