# Supplementary material for: “If I’ve got latent TB, I would like to get rid of it”: Derivation of the CARD (Constraints, Actions, Risks, and Desires) Framework informed by South African healthcare worker perspectives on latent tuberculosis treatment
Source: PLoS One. 2021 Aug 18;16(8):e0254211. doi: 10.1371/journal.pone.0254211 (PMC8372902; doi:10.1371/journal.pone.0254211)
Supplement: Attachment — Submitted filename: PLOS ONE_Latent TB Review.docx [file pone.0254211.s002.docx]

**PLOS ONE**

**“If I've got latent TB, I would like to get rid of it”: Derivation of the CARD (Constraints, Actions, Risks, and Desires) Framework informed by South African healthcare worker perspectives on latent tuberculosis treatment.**

Thank you for the opportunity to read and review your manuscript. I particularly enjoyed reading the manuscript, as it is a qualitative study looking at latent TB, which is particularly relevant and a large problem in South Africa. Overall, the author has made compelling points from the perspective of Healthcare workers. However, some revisions are needed. Overall, it is recommended that the author goes back to the manuscript to rephrase or break up certain sentences that are long. Fixing the structure of the sentences will help the overall flow or provide clarity in their statements. It would also be helpful if the author gave some further clarity on some of the challenges or limitations with TB screening given the nature of the occupational health systems, as per the available literature, to further support this statement provided in the introduction. The author makes use of the COREQ guidelines, offering additional detail on the qualitative methodology used. The author also uses an analytical framework which guides and structures the analysis process and results. Thank you for using an innovative and creative approach (as per Figure 1 and Table 1), to address the gaps and issues as discussed in this manuscript.

Additional revision recommendations have been listed below;

ABSTRACT

- Line 13: Consider changing starting the sentence with ‘we did’, as this currently sounds more informal, to ‘we conducted’.
- Line 20-21: Rephrase; Despite LTBI knowledge gaps and safety and cost concerns’ to ‘Despite LTBI knowledge gaps, safety and cost concerns’

INTRODUCTION

- Line 88-89: This statement may come through stronger if more clarity was provided
- Line 83-97: Suggestion would be to rephrase this sentence to provide further clarity

METHODS

- Line 115-118: ‘For this qualitative study, participants were purposively sampled to achieve systematic representation of a variety of perspectives and have 50% of participants with a positive baseline LTBI result (based on Quantiferon-Plus testing) that was known to both participant and researcher’. Considering including and ‘to have 50%’ so the sentence flows better.
- As per the COREQ guidelines, could you provide more information on the interviewers training? Were they part of the parent study, or were they unfamiliar to the participants?
- Line 151-154 needs rephrasing
- Line 170 – is missing a ‘,’ after context
- The author indicates, ‘To interpret HW perspectives in context our initial coding included four main themes: risk perception, regarding occupational TB, perceptions related to HWs’ duty of care to patients, TB-IC, and individual versus hospital responsibility for HW protection’, how were these themes derived? Could the author operationalize this process and how the themes were selected/emerged?
- Line 164: Please introduce here that the section pertains to the initial analysis that guided the development of the CARD framework, as initially it seems like the author is already presenting some results, which are then later applied to the data for analysis.
- As per the COREQ guidelines, were transcripts returned to participants for review? Did any participants receive a reimbursement or incentive? How was interrater reliability maintained or final presented themes evaluated amongst the team?

RESULTS

- The author presents a statement and a quote for each topic, under each theme. It would strengthen the manuscript, if the author offered some clarification or interpretation of the statements and quotations, to strengthen and further represent the sub-theme.
- Typo: Line 250 missing a full-stop at the end
- Could the author unpack or provide more clarity to the following statement: ‘Many reported discomfort, stigma, and potential negative impact on the clinician-patient relationship made it challenging to wear either a mask or a N95 respirator for prolonged periods’.
- Consider rephrasing line 289 – 291.
- Line 374-377: Consider breaking into smaller sentences.

DISCUSSION

- Line 439 – 442: The ‘social science informed network model of human behavior’ was not mentioned in the methodology until in the discussion.
- Line 460 – 462: Requires rephrasing
- Line 469 – 475: How are these conclusions regarding the tool derived from the data presented?
- Line 488 – 492: This longer sentence needs to be broken up; lacks clarity
